# Hemin as a novel candidate for treating COVID-19 via heme oxygenase-1 induction

**DOI:** 10.1038/s41598-021-01054-3

**Published:** 2021-11-02

**Authors:** Dong-Hwi Kim, Hee-Seop Ahn, Hyeon-Jeong Go, Da-Yoon Kim, Jae-Hyeong Kim, Joong-Bok Lee, Seung-Yong Park, Chang-Seon Song, Sang-Won Lee, Sang-Do Ha, Changsun Choi, In-Soo Choi

**Affiliations:** 1 Department of Infectious Diseases, College of Veterinary Medicine, Konkuk University, 120 Neungdong-ro, Gwangjin-gu, Seoul 05029 Republic of Korea; 2 Advanced Food Safety Research Group, BrainKorea21 Plus, Chung-Ang University, Anseong, Gyeonggi 17546 Republic of Korea; 3 Department of Food and Nutrition, School of Food Science and Technology, Chung-Ang University, Anseong, Gyeonggi 17546 Republic of Korea

**Keywords:** SARS-CoV-2, Antimicrobial therapy

## Abstract

Severe acute respiratory syndrome coronavirus 2 (SARS-CoV-2) is the causative agent of the coronavirus disease-19 (COVID-19). More than 143 million cases of COVID-19 have been reported to date, with the global death rate at 2.13%. Currently, there are no licensed therapeutics for controlling SARS-CoV-2 infection. The antiviral effects of heme oxygenase-1 (HO-1), a cytoprotective enzyme that inhibits the inflammatory response and reduces oxidative stress, have been investigated in several viral infections. To confirm whether HO-1 suppresses SARS-CoV-2 infection, we assessed the antiviral activity of hemin, an effective and safe HO-1 inducer, in SARS-CoV-2 infection. We found that treatment with hemin efficiently suppressed SARS-CoV-2 replication (selectivity index: 249.7012). Besides, the transient expression of HO-1 using an expression vector also suppressed the growth of the virus in cells. Free iron and biliverdin, which are metabolic byproducts of heme catalysis by HO-1, also suppressed the viral infection. Additionally, hemin indirectly increased the expression of interferon-stimulated proteins known to restrict SARS-CoV-2 replication. Overall, the findings suggested that HO-1, induced by hemin, effectively suppressed SARS-CoV-2 in vitro. Therefore, HO-1 could be potential therapeutic candidate for COVID-19.

## Introduction

Since the coronavirus disease-19 (COVID-19) outbreak was first reported in Wuhan, China, more than 143 million cases have been reported, and currently, the global death rate is 2.13%^1^. COVID-19 is caused by the severe acute respiratory syndrome coronavirus 2 (SARS-CoV-2), and the major symptoms of COVID-19 include fever, dry cough, chest pain, and fatigue, whereas headaches, dizziness, abdominal pain, and diarrhea are also observed in rare cases^2^. To date, antiviral drugs effective against human immunodeficiency virus (HIV), influenza virus, Ebola virus, hepatitis virus, cytomegalovirus (CMV), and herpes simplex virus have been used in attempts to treat COVID-19; however, no specific drug has been approved for the treatment of COVID-19 due to the variable treatment effects on the virus^2^. Therefore, owing to the rapid spread of COVID-19 worldwide, it is necessary to not only develop new vaccines but also to consider whether previously known antiviral agents can suppress SARS-CoV-2 replication^3^.

Heme oxygenase-1 (HO-1), encoded by *Hmox1*, is a cytoprotective enzyme that inhibits the inflammatory response and reduces oxidative stress^4^. HO-1 expression can be induced by hemin, CoPP-9, and andrographolide^5–7^. HO-1 catalyzes heme into carbon monoxide, biliverdin, and iron, which are also known to inhibit the proliferation of various viruses^8^. HO-1 was also shown to interact with interferon (IFN) regulatory factor 3, independent of its enzyme activity, which activated the type I IFN response to inhibit infection by influenza A virus^9^. Hemin, which is the representative HO-1 inducer, has antiviral effects against several viruses including hepatitis A virus^5,10–12^. Induction of HO-1 with other chemicals demonstrates the antiviral activity against hepatitis B and C viruses, Ebola virus, HIV, dengue virus, Zika virus, and human respiratory syncytial virus^13,14^. The suitability of HO-1 as an antiviral candidate for SARS-CoV-2 has been discussed in recent studies^15,16^.

In this study, we primarily used hemin to induce HO-1 expression, which led to a reduction in SARS-CoV-2 replication in vitro. We also attempted to identify the basic mechanisms underlying the antiviral effects of HO-1, with respect to its enzymatic as well as non-enzymatic functions.

## Results

### HO-1 induction by hemin and anti-SARS-CoV-2 activity

**Figure 1:**
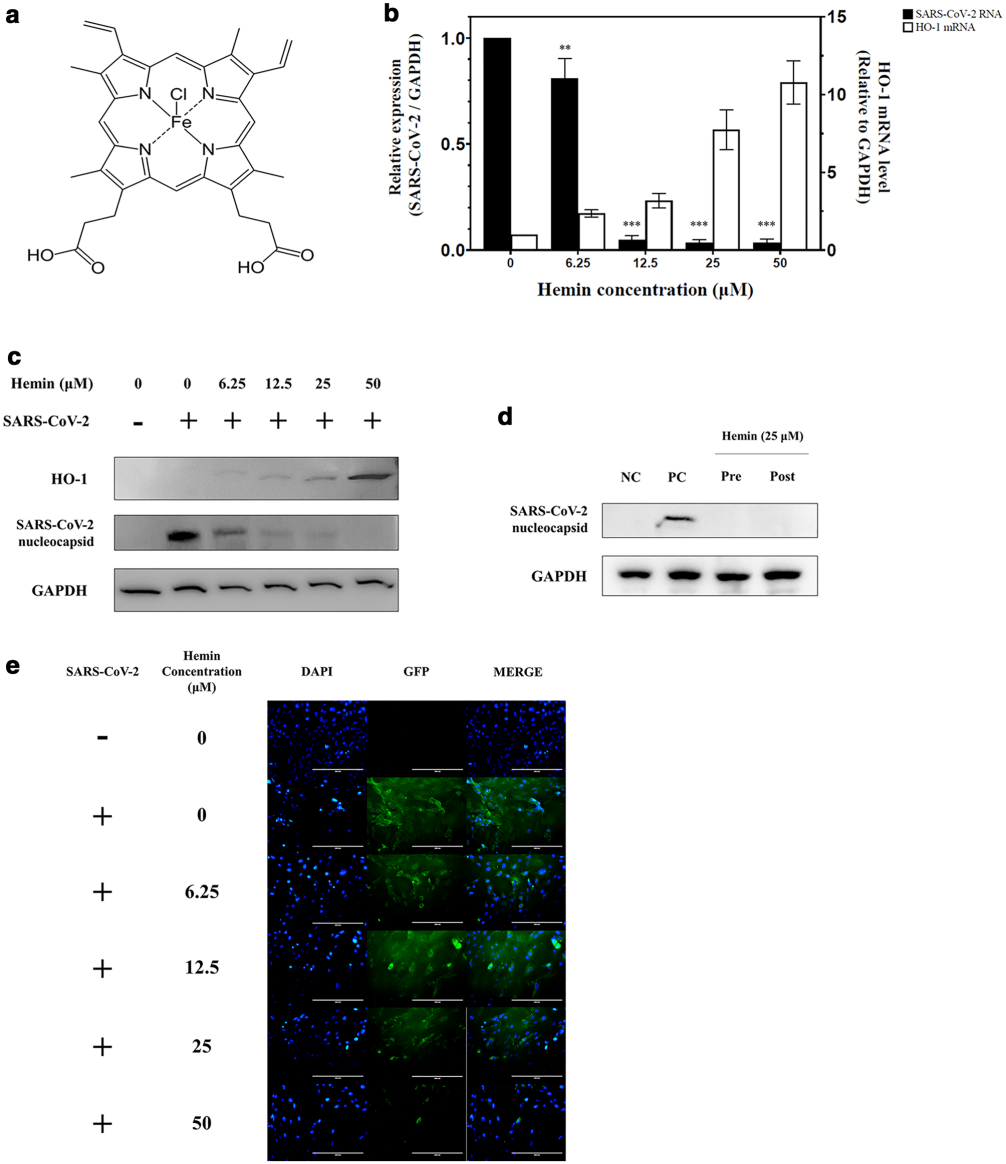
Changes in the induction of SARS-CoV-2 RNA and HO-1 expression in Vero76 cells at different concentrations of hemin. (**a**) Structure of hemin. The molecular formula of hemin is C_34_H_32_ClFeN_4_O_4_. (**b**) Vero76 cells infected with the SARS-CoV-2 strain NCCP43326 (multiplicity of infection (MOI): 0.001) were treated for 24 h without or with hemin at various concentrations. As the hemin concentration increased, the HO-1 mRNA expression increased, as expected, and the SARS-CoV-2 RNA expression decreased concomitantly. (**c**) Changes in the expression of HO-1 and SARS-CoV-2 nucleocapsid protein at different hemin concentrations. As the concentration of hemin increased, the width of the HO-1 protein band was observed to increase, and the viral protein content decreased. Comparison with the levels of GAPDH, a housekeeping protein, showed that equal quantities of cells were used in this experiment (also see Supplementary Fig. S4a). (**d**) Time-of-addition experiment with hemin. In the pre-treatment group (Pre), Vero76 cells were pre-treated with hemin for 1 h, and then infected with SARS-CoV-2. In the post-treatment group (Post), the cells were treated with hemin for 2 h after SARS-CoV-2 infection; NC (negative control): MOCK control, PC (positive control): only infected with SARS-CoV-2. (also see Supplementary Fig. S4b) (**e**) Cellular distribution of SARS-CoV-2 spike proteins. Vero76 cells pre-treated with hemin at various concentrations were infected with SARS-CoV-2 (MOI: 0.001). Immunofluorescence assay (IFA) for SARS-CoV-2 spike protein was conducted 24 h post-infection; green fluorescence: SARS-CoV-2 spike protein; blue fluorescence: DAPI staining for nuclei. Results of the IFA were analyzed using fluorescence microscopy. Original magnification: × 200. Data are presented as the mean ± standard deviation of at least three independent experiments. **P < 0.01, ***P < 0.001.

Vero76 cells were pretreated with hemin at various concentrations for 1 h and then infected with SARS-CoV-2. The cells were harvested at 24 h post-infection (hpi), and the intracellular viral RNA or viral protein content was analyzed. As expected, pre-treatment with hemin (Fig. 1a) successfully increased the HO-1 mRNA and protein content in a dose-dependent manner (Fig. 1b, c). Hemin was also shown to exhibit antiviral activity against SARS-CoV-2. Hemin induced the depletion of the SARS-CoV-2 RNA and viral nucleoprotein content in a dose-dependent manner (Fig. 1b, c). Therefore, the gradual increase in hemin concentration led to an increase in HO-1 mRNA and protein synthesis and the concomitant depletion of SARS-CoV-2 RNA and nucleocapsid protein (Fig. 1b, c). Time-of-addition experiments were performed to determine the time point at which viral replication was suppressed. The effects in the group pre-treated with hemin 1 h prior to the viral infection were monitored to determine whether viral infection was suppressed at the entry stage. The effects in the post-treatment group infected with the virus and then treated with hemin for 2 hpi were monitored to determine whether viral replication was suppressed during the intracellular replication stage after viral entry into the cells. The suppression of viral replication was observed in both groups, indicating that hemin treatment could inhibit the virus at the intracellular replication stage (Fig. 1d). The immunofluorescence assay (IFA) (Fig. 1e) showed that the number of cells containing the SARS-CoV-2 spike protein decreased as the hemin concentration increased at 24 hpi.

### Cytotoxicity and effective hemin concentration in vitro

**Figure 2:**
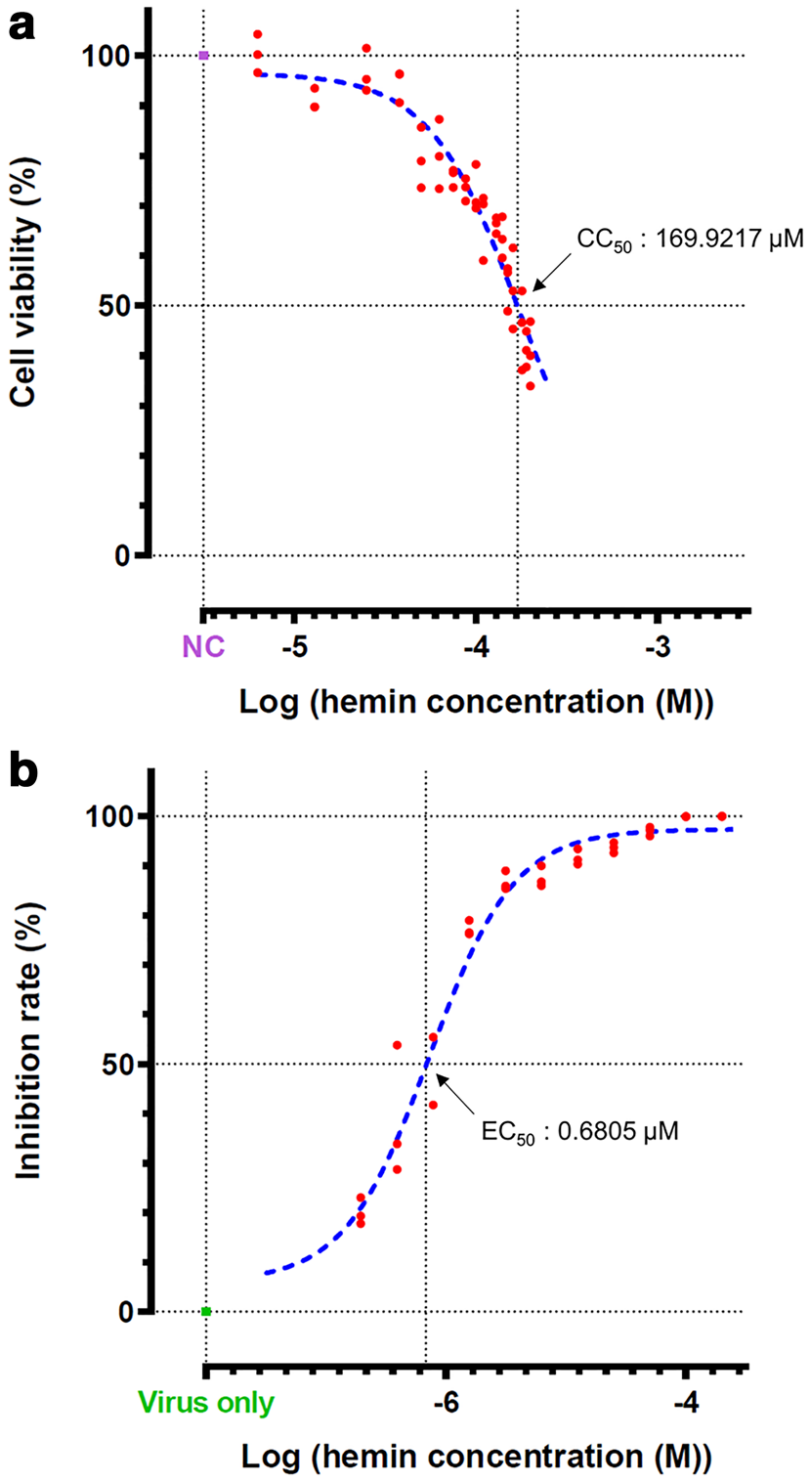
Pharmacological properties of hemin. (**a**) Viability percentages (compared with that of hemin-untreated cells) of Vero76 cells and (**b**) rate of SARS-CoV-2 inhibition at 72 h post-treatment with the indicated concentrations of hemin. Data are presented as the mean ± standard deviation of at least three independent experiments.

Since hemin exerted an antiviral effect against SARS-CoV-2, we conducted cytotoxicity assays to confirm whether the reduction in viral load was attributed to its cytotoxicity. Serial dilutions of hemin were applied to the cells, and cell viabilities were compared 2 days later. Based on the cytotoxicity results, the 50% cytotoxicity concentration (CC_50_) of hemin was calculated to be 169.9217 μM (Fig. 2a). The effective concentration at 50% (EC_50_) was calculated in parallel with cytotoxicity by measuring the viral RNA titers in the supernatants 2 days post-infection (dpi). The EC_50_ of hemin for suppressing viral growth was 0.6805 μM (Fig. 2b). The selectivity index (SI) of hemin was approximately 249.701 (Table 1). These results indicate the safety and effectiveness of hemin as a therapeutic agent.

**Table 1:** Cytotoxic concentration (CC_50_) and effective concentration (EC_50_) of hemin in Vero76 cells against the SARS-CoV-2 strain NCCP43326.

Virus	Vero76 cells CC_50_: 169.9217 μM	
	EC_50_	SI^a^
SARS-CoV-2 virus (NCCP43326)	0.6805 μM	249.7012

^a^SI: selectivity index (CC_50_/EC_50_).

### HO-1 overexpression attenuates SARS-CoV-2 replication

**Figure 3:**
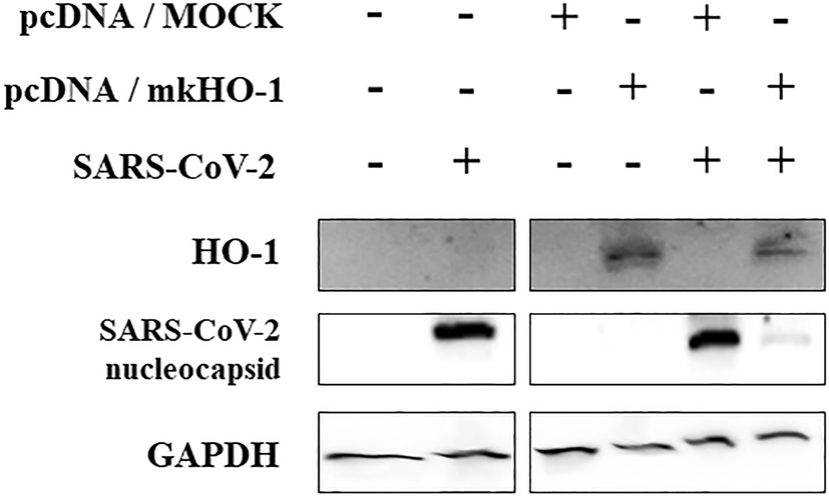
HO-1 overexpression inhibited SARS-CoV-2 proliferation. Vero76 cells were transfected with the monkey HO-1 gene cloned into the pcDNA 3.1(+) vector or empty pcDNA 3.1(+) vector control. GAPDH expression was monitored to confirm that equal quantities of cells were loaded (also see Supplementary Fig. S4c).

The pcDNA 3.1(+) vector harboring the monkey HO-1 gene (GeneBank accession number: XM028827760) was constructed using gene synthesis for transiently expressing exogenous HO-1 (pcDNA/mkHO-1). The pcDNA/mkHO-1 and empty pcDNA 3.1(+) vector (pcDNA/MOCK) were transfected to Vero76 cells. Monkey HO-1 proteins were expressed in the Vero76 cells under a CMV promoter in pcDNA/mkHO-1. Two days after the transfection, the cells were infected with SARS-CoV-2. The cells were harvested at 24 hpi, and HO-1 protein expression was measured. As shown in Fig. 3, HO-1 was clearly detected in the cells transfected with pcDNA/mkHO-1 but not in the ones transfected with pcDNA/MOCK. As observed in previous results, the transient overexpression of HO-1 inhibited SARS-CoV-2 replication.

### Metabolites from heme catalysis by HO-1 exhibit antiviral effects against SARS-CoV-2

**Figure 4:**
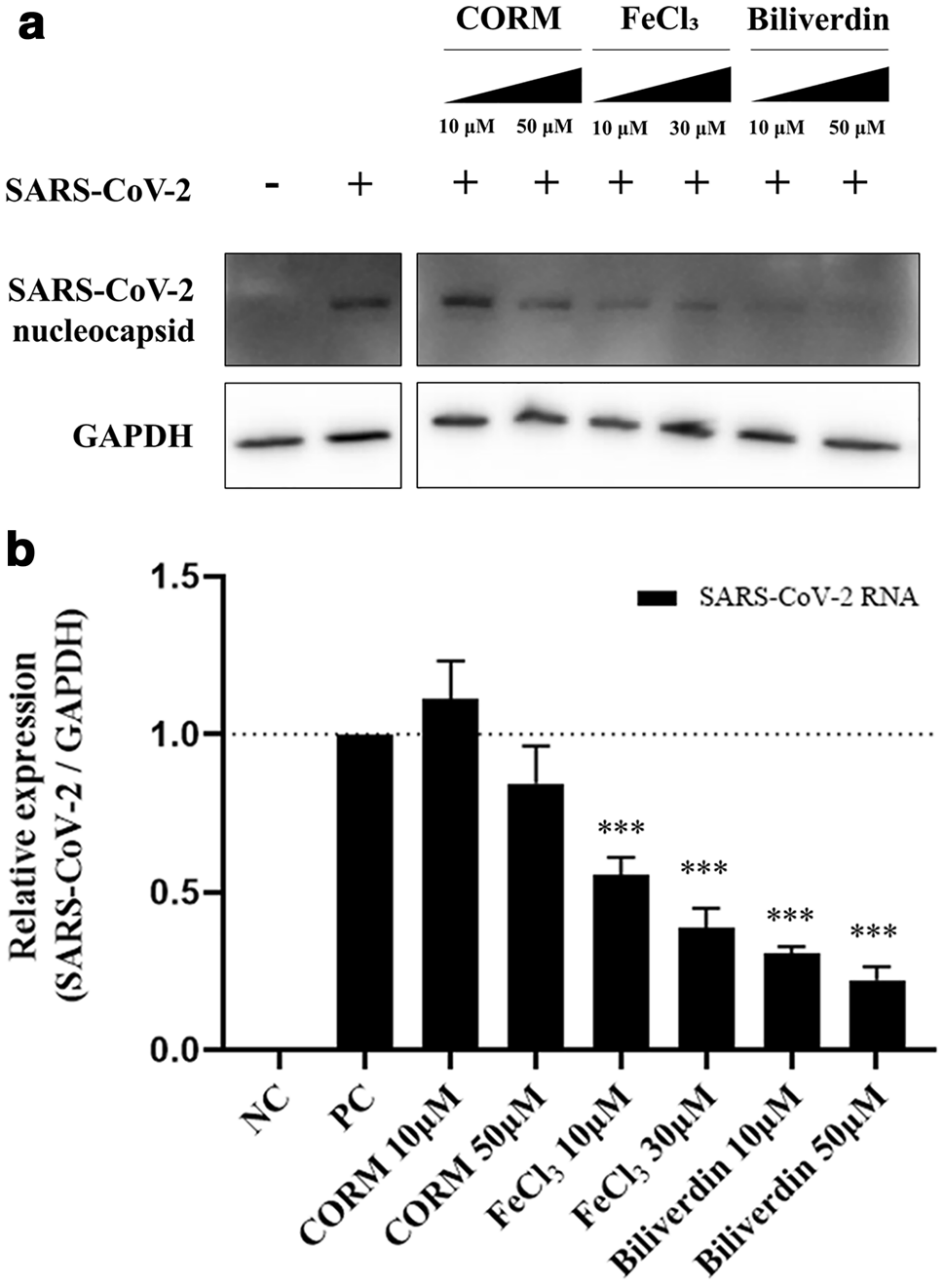
Antiviral efficacy of the metabolites produced by HO-1. The inhibition of SARS-CoV-2 proliferation by carbon monoxide, iron ions, and biliverdin produced by HO-1 was confirmed. Treatment with CORM (releases carbon monoxide), FeCl_3_, and biliverdin was performed 1 h before SARS-CoV-2 infection. At 24 h post-infection, (**a**) western blot assay (also see Supplementary Fig. S4d) and (**b**) qPCR were performed to compare the changes in the viral protein and RNA contents. ***P < 0.001.

The catalysis of heme by HO-1 is known to yield free iron, biliverdin, and carbon monoxide (CO) as metabolites^17^. We treated Vero76 cells with a CO-releasing molecule (CORM, induces CO production), FeCl_3_ (induces free iron production), and biliverdin. The treated cells were infected with SARS-CoV-2, and the replication of the virus was assessed. Immunoblotting and qPCR results demonstrated that FeCl_3_ and biliverdin significantly suppressed SARS-CoV-2 RNA and nucleocapsid protein expression in a dose-dependent manner, whereas CORM did not (Fig. 4a, b). Additionally, cytotoxicity of the three chemicals CORM, FeCl_3_, and biliverdin were not observed to Vero76 cells (Supplementary Fig. S1). These results suggest that free iron and biliverdin derived from heme catalysis by HO-1 could suppress the replication of SARS-CoV-2.

### Enzyme-activity-independent antiviral effect of HO-1 against SARS-CoV-2

**Figure 5:**
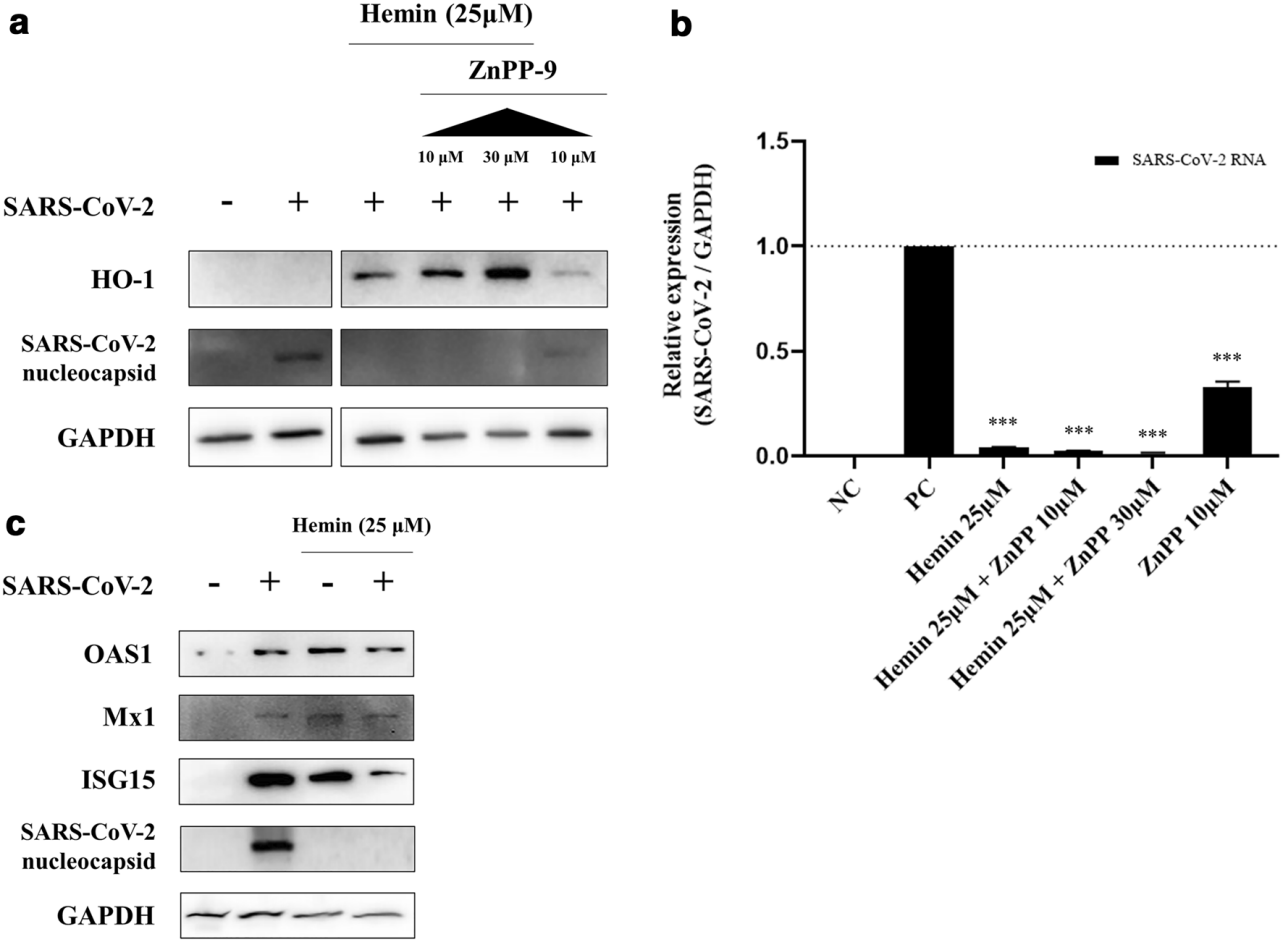
Antiviral efficacy via ISG induction, and not enzymatic action of hemin. Effect of ZnPP-9 (selective inhibitor of HO-1 enzyme activity) on SARS-CoV-2 infection. Vero76 cells were treated with/without hemin or ZnPP-9. The concentration of hemin was 25 μM. ZnPP-9 did not reverse the antiviral effects of HO-1 induced by hemin. (**a**) ZnPP-9 induced HO-1 protein expression even in the absence of hemin (also see Supplementary Fig. S4e), and (**b**) HO-1 induction also suppressed the replication of the virus. (**c**) ISG induction by SARS-CoV-2 and hemin (also see Supplementary Fig. S4f, g). The expression of ISG proteins could be induced only under SARS-CoV-2 infection; however, the ISG proteins could not completely suppress viral replication. Treatment with hemin also induced the expression of ISG proteins and inhibited the replication of the virus. ***P < 0.001.

To determine if the antiviral activity of HO-1 was associated exclusively with its enzymatic activity, the cells were treated with zinc protoporphyrin-9 (ZnPP-9), an inhibitor of HO-1 enzymatic activity, along with hemin or alone. A greater reduction in SARS-CoV-2 RNA and protein content was observed when the cells were treated simultaneously with hemin and ZnPP-9 than with hemin alone (Fig. 5a, b). Conversely, transient knock-down of HO-1 expression with short interfering RNA (siRNA) specific to HO-1 significantly abolished the antiviral effect of HO-1 on SARS-CoV-2 (Supplementary Fig. S2). These results indicate that the inhibition of the enzymatic activity of HO-1 does not reduce the antiviral activity of HO-1. In addition, we observed low levels of HO-1 expression and suppression of SARS-CoV-2 replication in cells treated with 10 μM ZnPP-9 alone (Fig. 5a, b). Existing research shows that ZnPP-9 attenuates HO-1 enzymatic activity but increases expression of HO-1 protein. The elevated HO-1 protein by ZnPP-9 indirectly could promote the antiviral state through the IFN pathway^8,18^. We confirmed that hemin-induced HO-1 expression could promote the expression of IFN-stimulated gene (ISG) proteins (OAS1, Mx1, and ISG15). The production of OAS1, Mx1, and ISG15 proteins increased in the SARS-CoV-2 infected cells, hemin-treated cells, and cells infected with SARS-CoV-2 after hemin treatment (Fig. 5c). While the three ISG proteins were produced in cells infected with SARS-CoV-2 in the absence of hemin, the viral nucleocapsid protein was detected (Fig. 5c). However, the three ISG proteins were expressed and the viral nucleocapsid protein was not detected in the cells infected with SARS-CoV-2 and treated with hemin (Fig. 5c). This indicates that ISGs produced under induction by the virus could not sufficiently suppress the replication of SARS-CoV-2.

## Discussion

Several antiviral therapeutics are currently used for viral infections, and some of them have been found to be effective against COVID-19^2,19^. Studies on the antiviral effects of viral RNA polymerase inhibitors (remdesivir/favipiravir), viral protein synthesis inhibitors (ritonavir/lopinavir), inhibitors of viral entry (hydroxychloroquine/chloroquine), and immunomodulators (nitazoxanide/ivermectin) are underway; however, the antiviral effects of HO-1, particularly against SARS-CoV-2, are yet to be investigated thoroughly^19^. HO-1 is known to exhibit cytoprotective and anti-inflammatory activities through its byproducts, and these actions may help modulate inflammation in various diseases^20^. The immunomodulatory and antiviral properties of HO-1 against respiratory viruses, including influenza virus and respiratory syncytial virus, have been reported^8^. A recently published research article hypothesized that hemin-induced HO-1 may control SARS-CoV-2 infection by stimulating an anti-inflammatory pathway^16^. However, the authors did not provide evidence of the antiviral activity of HO-1 against SARS-CoV-2.

In this study, we first demonstrated that HO-1 could suppress the replication of SARS-CoV-2 in an in vitro system. We used the efficient HO-1 inducer hemin, an iron-containing porphyrin which is widely known to induce HO-1 expression with a low rate of side effects^21,22^. Our findings indicated that the treatment of cells with hemin at 6.25 μM (*P* < 0.01) and at concentrations above 12.5 μM (*P* < 0.001) effectively induced HO-1 expression and significantly suppressed viral replication within 24 hpi. Similar antiviral results were observed when CoPP-9 or andrographolide was used to treat the virus-infected cells. The fact that viral protein expression was suppressed even when the cells had been treated with hemin after viral entry indicates that hemin may be able to suppress viral replication at the intracellular replication phase, similar to remdesivir^23^. However, a recent study suggested that HO-1 induced by hemin could not inhibit SARS-CoV-2 infection^24^. They demonstrated the unchanged viral RNA levels from the cell culture supernatants whereas we determined reduction of both viral RNA and viral protein in the cells after treatment with hemin. These contradictory results between our and their studies might be caused by the different experimental conditions and methods.

Hemin is known to exert cytoprotective effects and low cytotoxicity. Commercially, hemin is available as PANHEMATIN®, which was approved by the US Food and Drug Administration in 1983, and its safety is guaranteed^21,22^. In this study, the CC_50_ value of hemin, which is an indicator of its cytotoxicity, was approximately 169.9217 μM, and the EC_50_ value, which represents the concentration of the agent that reduces the viral RNA load by 50%, was approximately 0.6805 μM. The CC_50_ value was approximately 249.7012 times higher than the EC_50_ value, and the SI was 249.7012. Compared with that of other FDA-approved chemicals, the SI of hemin indicates its safety and effectiveness as a therapeutic agent^25^.

To determine the specificity of HO-1 in its antiviral activity against SARS-CoV-2, we overexpressed monkey HO-1 through an expression vector. HO-1 was successfully expressed in the Vero76 cells transfected with the expression vector encoding the monkey HO-1 gene, and the suppression of SARS-CoV-2 replication in the cells was confirmed. This implies that the antiviral effect could be attributed to HO-1, and not solely to the porphyrin effect of hemin that blocks viral entry, as observed in case of HIV inhibition^26^. To investigate the mechanism underlying the antiviral effects of HO-1, we treated the cells with CORM, FeCl_3_, and biliverdin to evaluate the antiviral effects of the metabolic byproducts of heme decomposition by HO-1. These metabolites have been shown to exert antiviral effects on several viruses^27–29^. In our study, the treatment of cells with FeCl_3_ or biliverdin, but not CORM, significantly reduced the SARS-CoV-2 RNA and protein titers (*P* < 0.001), suggesting that the enzymatic action of HO-1 suppressed viral growth. However, when the cells were treated with hemin and ZnPP-9, a selective inhibitor of HO-1 enzyme activity, the suppression of SARS-CoV-2 replication was not reversed. These results are different from the knock-down experiment through siRNA, which inhibited the expression of HO-1 protein and reversed the suppression of SARS-CoV-2. This implies that the antiviral activity could be induced by other actions of HO-1 in addition to its enzymatic activity. While ZnPP-9 is known to inhibit the enzymatic action HO-1, it induced HO-1 protein expression, as shown in our results (Fig. 5a) ^18^. The expressed HO-1 indirectly helped establish the antiviral state by interacting with IFN regulatory factor 3, thereby promoting the expression of the ISGs OAS1, Mx1, and ISG15^8^. Type I IFNs (IFN-α and IFN-β) and type III IFNs (IFN-λ) provide potent antiviral immunity against SARS-CoV-2 via ISG protein expression^30–32^. However, SARS-CoV-2 replication is more rapid than the antiviral IFN responses at a higher viral load^33^. Under these conditions, the IFN responses cannot limit viral replication and may induce inflammatory responses, thereby causing tissue damage^31^. In this study, we demonstrated that while SARS-CoV-2 infection induced the expression of ISG proteins in the cells at 24 hpi, the proliferation of the virus was already complete (Fig. 5c). HO-1 can promote IRF3 phosphorylation and nuclear translocation, and the IRF3-mediated pathway appears to be associated with the earlier induction of ISG proteins and suppression of SARS-CoV-2 replication^6^.

In conclusion, hemin was found to be highly effective in controlling SARS-CoV-2 infection in vitro. The treatment of cells with hemin induced HO-1 expression, which suppressed SARS-CoV-2 replication directly through metabolites such as iron and biliverdin and indirectly via ISG proteins. Both antiviral mechanisms induced by HO-1 appear to inhibit viral growth in vitro. We believe these findings could contribute significantly to the development of therapeutic drugs for COVID-19.

## Methods

### Cell lines, viruses, and chemicals

Vero76 cells were obtained from the Korean Cell Line Bank and maintained in Dulbecco’s modified Eagle’s medium (DMEM) supplemented with 10% heat-inactivated fetal bovine serum (FBS, Gibco) in an atmosphere with 5% CO_2_ at 37 °C. The S strain of SARS-CoV-2 (NCCP43326) was obtained from the National Culture Collection for Pathogens. Vero76 cells were infected at a 0.001 multiplicity of infection of NCCP43326. The cells inoculated with the virus were maintained in DMEM supplemented with 2% FBS. Hemin, FeCl_3_, CORM, andrographolide, CoPP-9, and ZnPP-9 were purchased from Sigma-Aldrich, and biliverdin was purchased from Cayman Chemical Company.

### Cytotoxicity assay

The cytotoxicity of hemin, CORM-3, FeCl_3_, and biliverdin on Vero76 cells was assessed using the MTT assay. Vero76 cells were seeded in each well of a 96-well plate at 1 × 10^4^ cells/well. After 24 h, the cells were treated with hemin at a serial concentration for 48 h. After the supernatants were removed, MTT (5 mg/mL) and fresh serum-free media were added to the cells, followed by incubation in an atmosphere with 5% CO_2_ at 37 °C. After 3 h, 150 μL of dimethyl sulfoxide was added for solubilization. A spectrophotometer was used for plate reading to observe the viable cells at 540 nm. Cytotoxicity was analyzed in terms of the CC_50_ values obtained, using untreated cells as 100% viability controls for comparison.

### Reverse transcription-quantitative polymerase chain reaction (RT-qPCR)

Intracellular viral RNA and mRNA were extracted from the cell lysates using the RNeasy Mini RNA isolation kit (Qiagen), and viral RNA in the supernatant was extracted using the Patho Gene-spin DNA/RNA kit (Intron) according to the manufacturer’s instructions. RT-qPCR was performed using the One Step TB Green® PrimeScript™ RT-PCR Kit (Takara) with a Light Cycler instrument (Roche). The viral GE copy numbers were calculated from a standard curve generated using a plasmid DNA containing the M gene target sequence. The viral genomic copy numbers were divided by those of *GAPDH* to calculate the number of viruses contained per cell, and the relative HO-1 mRNA expression was analyzed using the 2^−ΔΔCt^ method. All experiments were repeated three times to obtain reliable results. The following primer sequences were used:

SARS-CoV-2 M gene: forward primer, 5′-GGYTCTAARTCACCCATTCA-3′; reverse primer, 5′-TGATACTCTARAAAGTCTTCATA-3′. *HO-1*: forward primer, 5′-CTTCAAGCTGGTGATGGC-3′; reverse primer, 5′-TGGAGCCGCTTCACATAG-3′. *GAPDH*: forward primer, 5′-GAAATCCCATCACCATCTTCCAGG-3′; reverse primer, 5′- GAGCCCCAGCCTTCTCCATG-3′.

### Transfection of short interfering RNA (siRNA)

For transient knock-down of HO-1, Vero76 cells were seeded in 6-well plates at 6 × 10^5^ cells/well and transfected with 30 pmol of HO-1-specific siRNA (AM16708, assay ID 11,152, Thermo Fisher Scientific) or control siRNA (AM4611, Thermo Fisher Scientific) using Lipofectamine RNAiMax transfection reagent according to the manufacturer’s instructions. One day after siRNA transfection, the cells were infected with SARS-CoV-2 or treated with 25 μM hemin.

### Western blot analysis

At 24 h post-inoculation, the cells were washed three times with PBS and harvested. Cell lysis was performed using 2× laemmli sample buffer (S3401, Sigma) and boiling at 95 °C. The cell lysates were then centrifuged (9000 × *g* for 1 min), and the supernatants were loaded on an SDS-polyacrylamide gel. After electrophoresis, the separated proteins were transferred to a nitrocellulose membrane and blocked overnight with 5% skim milk in PBS solution containing 0.05% Tween 20 (PBS-T) at 4 °C. The membranes were treated with an anti-HO-1 antibody (SAB1405949, Sigma), anti-GAPDH antibody (ab8245, Abcam), or anti-SARS-CoV-2 N antibody (GTX632269, GeneTex) in 2.5% skim milk-PBS-T for 1 h at room temperature. The membranes were then washed three times with PBS-T for 10 min in each round, treated for 1 h with a secondary antibody tagged with horseradish peroxidase in 2.5% skim milk-PBS-T solution, and washed three times with PBS-T for 10 min in each round. The protein bands were visualized using the SuperSignal™ West Pico PLUS Chemiluminescent Substrate (Sigma).

### IFA

IFA was conducted for the detection of SARS-CoV-2 spike proteins. At 24 hpi, the supernatants were removed, and the cells were fixed using 4% paraformaldehyde in PBS for 10 min at 25 °C. The fixed cells were washed three times with ice-cold PBS. To detect the intracellular viral proteins, the cells were permeabilized by treating with PBS containing 0.5% Triton X-100 for 10 min. After permeabilization, the cells were washed in PBS three times for 5 min in each round. The cells were then blocked with 1% bovine serum albumin (BSA) in PBS-T for 30 min at 25 °C. Next, an anti-SARS-CoV-2 spike protein antibody (GTX632604, GeneTex) was diluted in PBS-T with 1% BSA, and the blocked cells were treated using this as the primary antibody for 1 h in a humidified chamber at 25 °C. After the primary antibody reaction, the solution was decanted, and the cells were washed three times in PBS for 5 min in each washing step. For the secondary antibody reaction, the cells were treated with Alexa Fluor 488-conjugated anti-mouse IgG antibody in PBS-T with 1% BSA for 1 h at 25 °C in the dark. Following this, the solution was decanted, and the cells were washed three times in PBS for 5 min in each washing step. Counter staining was performed using 4′,6-diamidino-2-phenylindol.

### Statistical analysis

The experiments were conducted at least three times, and the data are expressed as the mean ± SD. The dose–response curves were plotted, and the Student’s *t*-test was performed using PRISM 8.0.1 (GraphPad Software). Differences were considered significant at *P* < 0.05.

## Supplementary Information

Supplementary Figures.

## Data Availability

All data generated or analyzed during this study are included in this published article (and its Supplementary Information file).
