# Supplementary material for: Hemin as a novel candidate for treating COVID-19 via heme oxygenase-1 induction
Source: Sci Rep. 2021 Nov 2;11:21462. doi: 10.1038/s41598-021-01054-3 (PMC8563742; doi:10.1038/s41598-021-01054-3)
Supplement: Supplementary file 1 — Supplementary Figures. [file 41598_2021_1054_MOESM1_ESM.docx]

**Supplementary Information**

**Supplementary Figures**

**
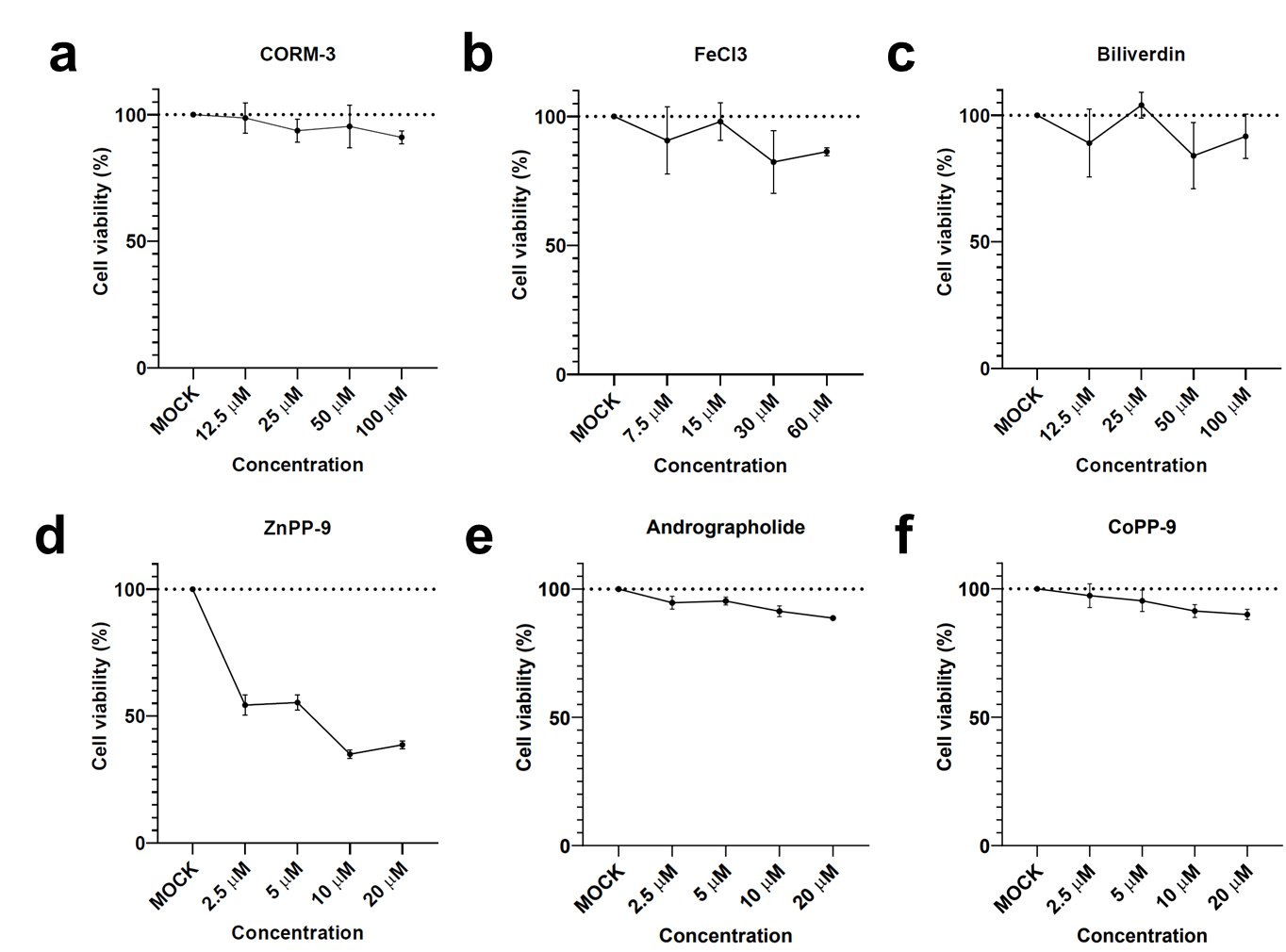
**

**Supplementary Fig. S1. In vitro cytotoxicity assays for chemicals used in this study.** Vero76 cells were incubated with chemicals used in this study for 48 hours to measure cytotoxicity. Cytotoxicity was determined by MTT assay for each chemical. Histograms represent the cell viability of Vero76 cells with respect to MOCK-treated cells (MOCK, 100%) after the treatment with (**a**) CORM-3, (**b**) FeCl_3_, and (**c**) biliverdin. Data are presented as the mean ± SD at least three independent experiments.


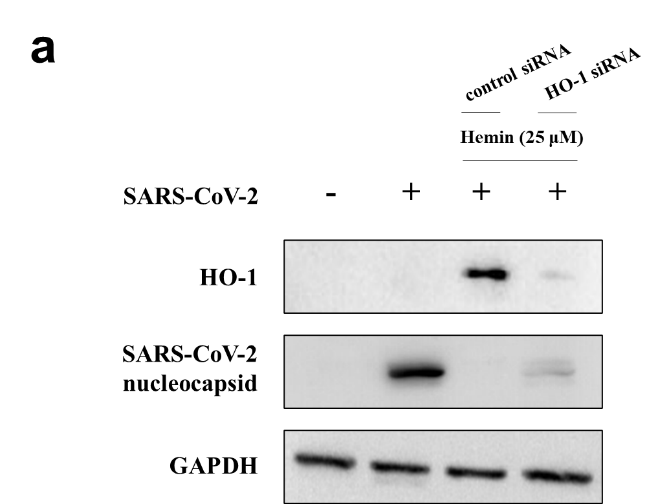

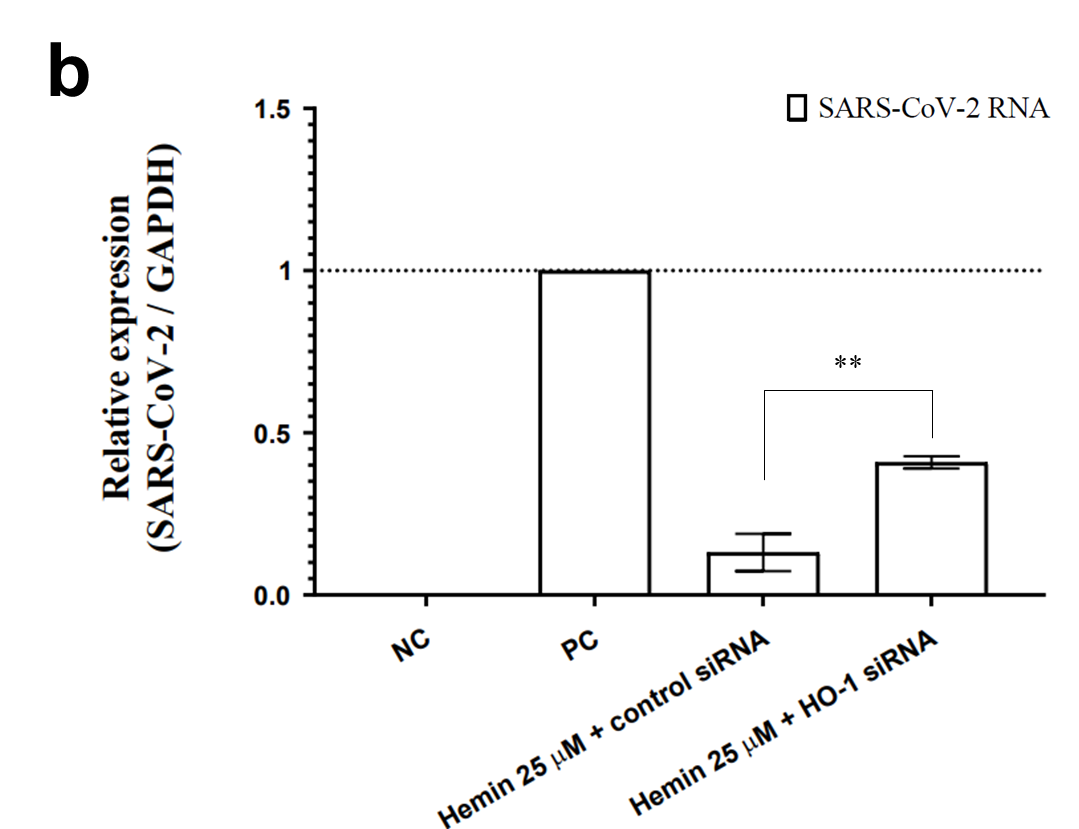


**Supplementary Fig. S2. Reversion of the hemin-induced antiviral effect against SARS-CoV-2 infection by HO-1 silencing.** Vero76 cells were transfected with HO-1-specific siRNA 1 day before the viral infection. Transfection of siRNA reduced HO-1 expression by hemin treatment and reversed the hemin-induced antiviral effect against SARS-CoV-2. (**a**) Viral protein and (**b**) viral RNA expression levels were reversed after HO-1 siRNA transfection (also see Supplementary Fig. **S4**h). Data are presented as the mean ± SD of at least three independent experiments. ***P*<0.01


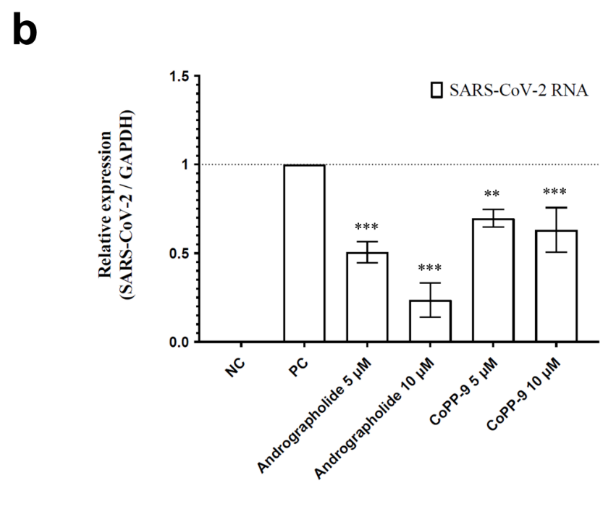

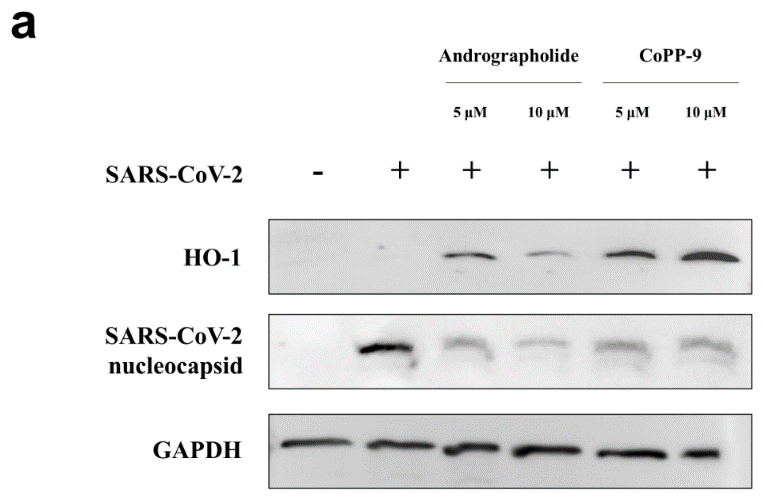


**Supplementary Fig. S3. Treatment with andrographolide or CoPP-9 suppresses SARS-CoV-2 infection.** The HO-1 inducers andrographolide and CoPP-9 exhibited antiviral activity against SARS-CoV-2. These chemicals induced HO-1 expression 24 h after treatment and suppressed the (**a**) viral protein and (**b**) viral RNA expression (also see Supplementary Fig. **S4**i). Data are presented as the mean ± SD of at least three independent experiments. ***P*<0.01, ****P*<0.001

**
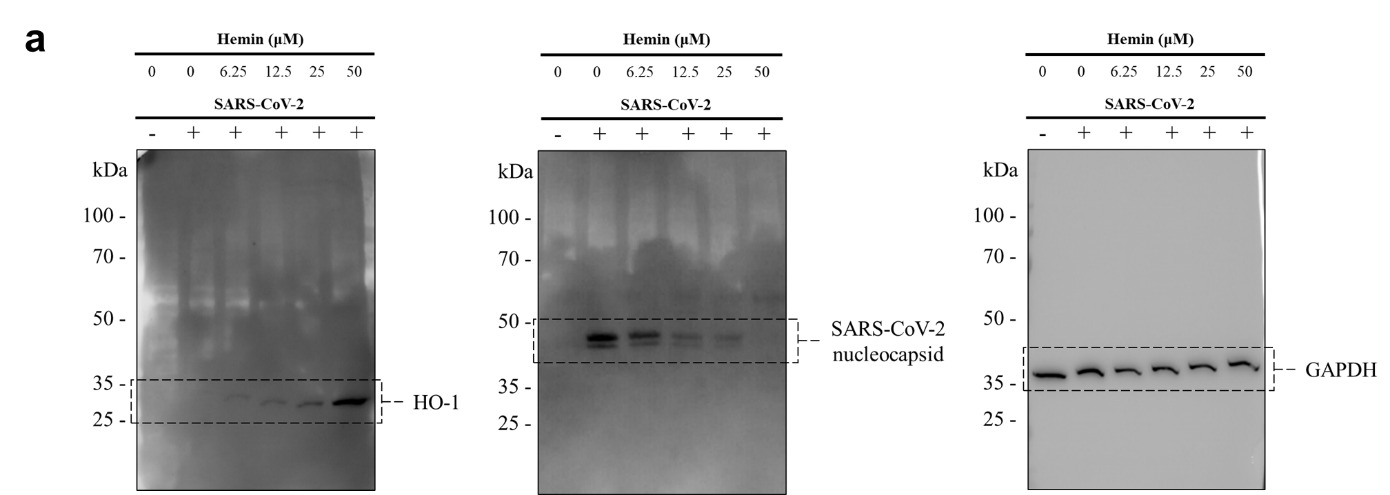
**

**
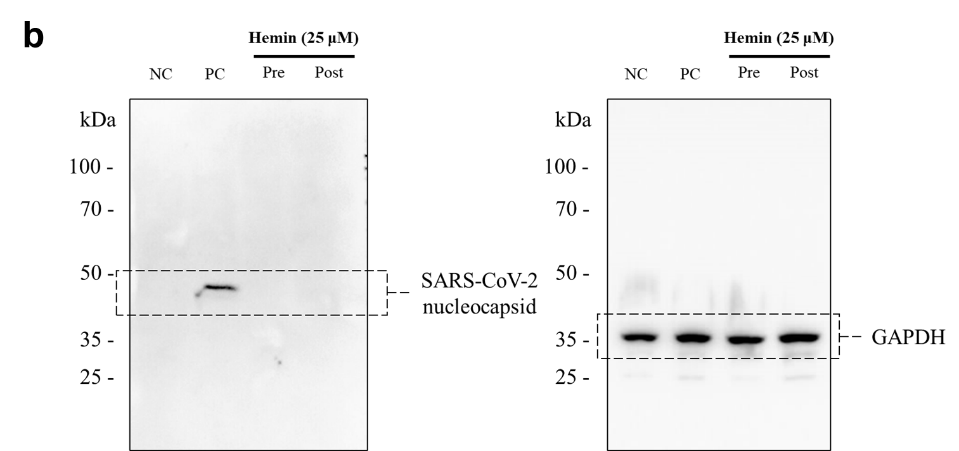
**

**
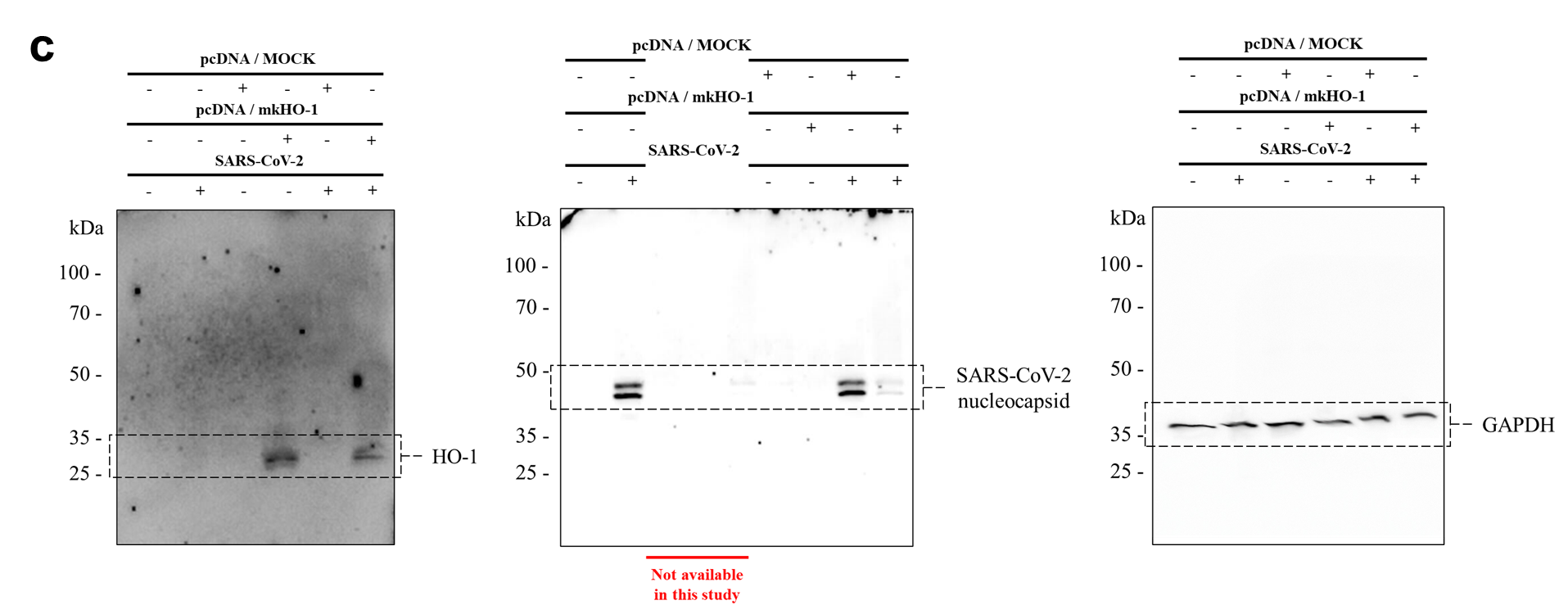
**

**
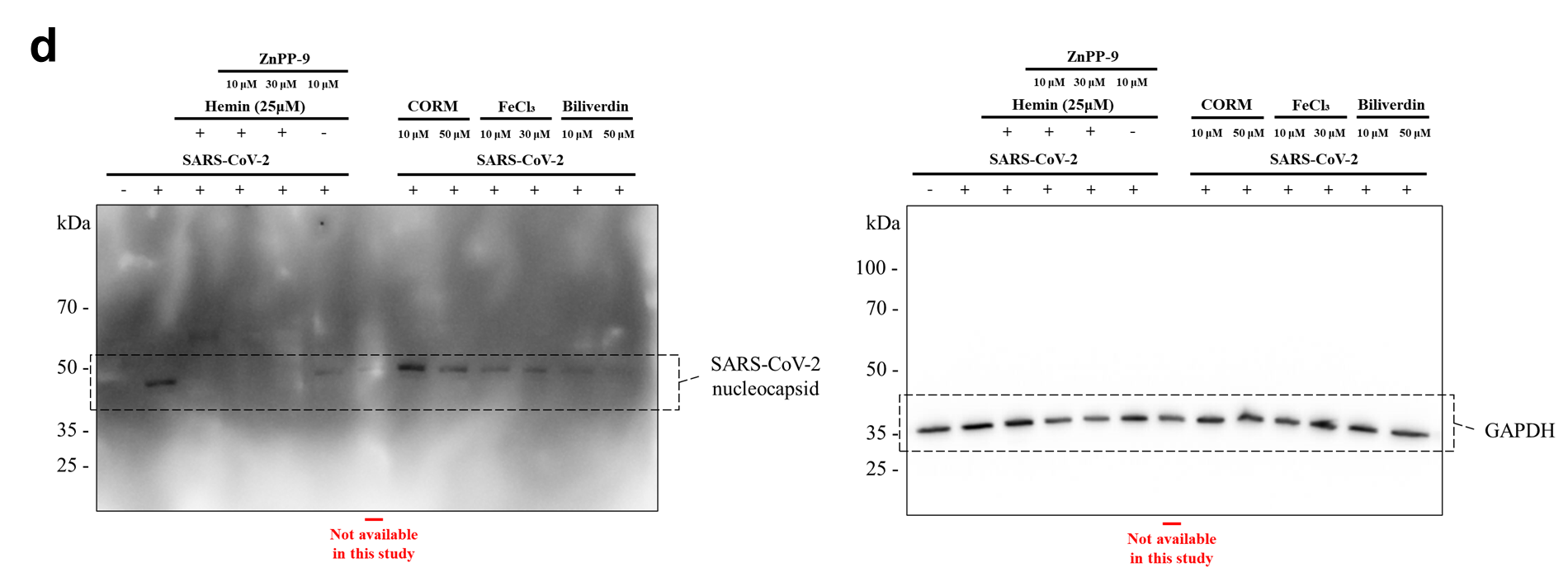
**

**
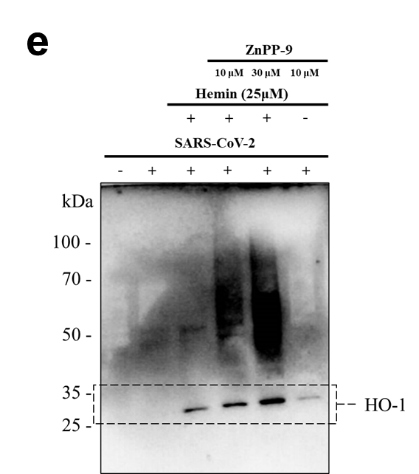

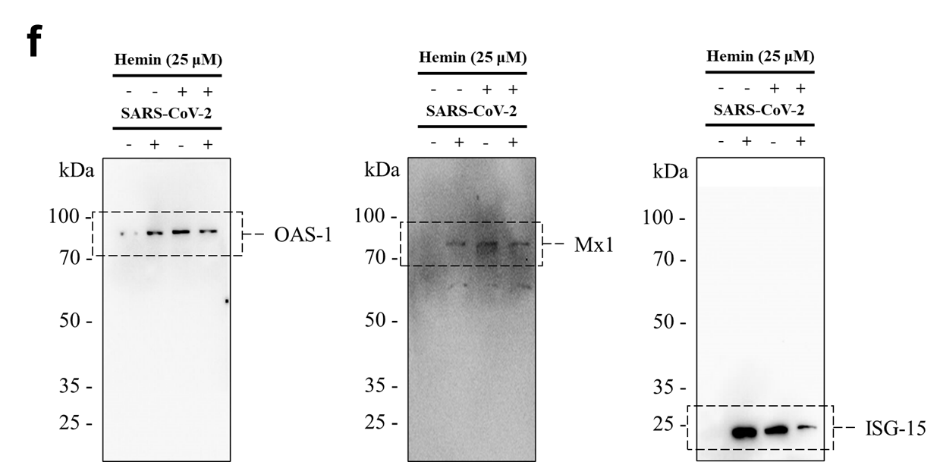
**

**
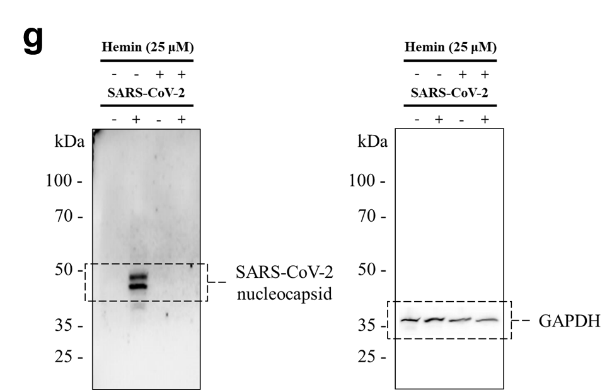
**

**
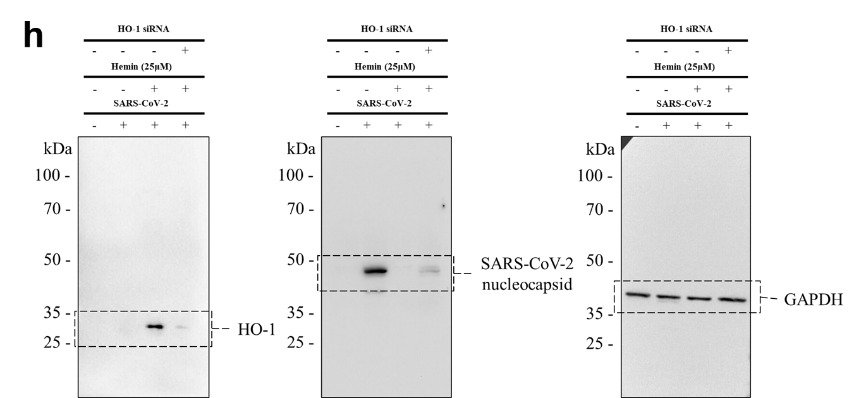
**

**
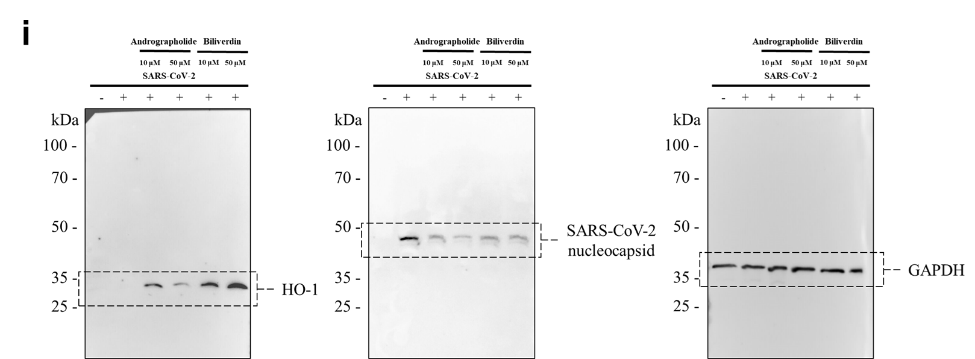
**

**Supplementary Fig. S4. Full-length Western blot images.** (**a**) Full-length image for figure **1**c, (**b**) full-length image for figure **1**d, (**c**) full-length image for figure **3**, (**d**) full-length image for figure **4**a and **5**a, (**e**) full-length image for figure **5**a, (**f, g**) full-length images for figure **5**c, (**h**) full-length image for Supplementary Fig. S**2**a, and (**i**) full-length image for Supplementary Fig. S**3**a.
